# Effect of Positive Psychological Intervention on Posttraumatic Growth among Primary Healthcare Workers in China: A Preliminary Prospective Study

**DOI:** 10.1038/srep39189

**Published:** 2016-12-20

**Authors:** Xin Xu, Mu-li Hu, Yu Song, Zhang-xiu Lu, You-qiao Chen, Da-xing Wu, Tao Xiao

**Affiliations:** 1 Medical Psychological Center, The Second Xiangya Hospital, Central South University, Changsha, Hunan 410011, China; 2 Research Office, The Second Xiangya Hospital, Central South University, Changsha, Hunan 410011, China; 3 Research Office, People’s Hospital of Hunan Province, Changsha, P. R. China

## Abstract

Posttraumatic growth (PTG) is defined as positive psychological change in the wake of highly challenging circumstances. Healthcare workers in particular are more vulnerable to stressors and trauma than the general population. The current study examined the use and effectiveness of a novel positive psychological intervention based on Chinese traditional culture to improve PTG in hospital healthcare workers. The intervention was provided to 579 healthcare workers at hospitals in Guilin, Shenzhen and Xiangtan. Scores on the Posttraumatic Growth Inventory (PTGI) and its subscales were significantly higher after intervention than before (p < 0.001). Of the five aspects of PTG, the aspect of “new possibilities” benefited the most from intervention (Cohen’s d = 0.45). PTG in women, nurses and college graduates increased to a greater extent than other participants after intervention. It was concluded that our novel intervention is effective at improving PTG in medical staff.

Devastating events, such as a severe disease, bereavement, a car accident, a natural disaster or other life-threatening challenges, have great impact on individuals. Prior studies have described how traumatic events can damage psychological health and quality of life leading to illnesses such as posttraumatic stress disorder (PTSD) and depressive symptoms^1^,^2^,^3^,^4^. In the 1990s, Martin Seligman initiated the positive psychology movement, advocating the study of positive psychological traits and focusing on well-being along with potential development^5^. Due to the development of positive psychology, increasing studies have explored the possible positive consequence after trauma^6^,^7^,^8^. The most common salutary response to trauma is posttraumatic growth (PTG). The definition of PTG is widely accepted as “positive psychological change in the wake of struggling with highly challenging life circumstances”^9^. The positive change is seen as improved psychological health after the occurrence of a crisis and the improvement has a profound impact on the survivor^9^. PTG is different from resilience. Contrary to resilience, which emphasizes an adaptive response to an adverse situation which results in sustained physical functioning^10^, PTG stresses a higher level of functioning, surpassing pre-trauma functioning^11^. Perceived benefits from crisis can be divided into five aspects: relating to others, new possibilities, personal strength, spiritual change and appreciation of life^12^.

Traumatic experience does not directly cause PTG but is rather a catalyst to meaning-making which is a necessary step in changing old (negative) schemas to a more positive perspective.

PTG arises from a cognitive restructuring and as such, a number of factors affect the development of PTG. Rumination is a necessary cognitive process in changing one’s worldview after suffering from a traumatic event and is therefore considered as a key factor in the development of PTG^13^. Along with rumination, resilience, which can decrease pathogenic reactions after trauma, is an important factor in the development of PTG^14^. Social support and the ability to share traumatic experiences and feelings with family and intimate friends have a positive effect on PTG^15^. Social interaction can promote the development of new schemas as well as providing a modified and meaningful narrative about the traumatic experience^16^. In addition, positive emotions are closely associated with PTG^17^. Under a positive emotional state, individuals are more likely to broaden their attention and cognitive competence, which in turn facilitates PTG^18^. Lastly, positive coping strategies, both problem-focused coping strategies and emotion-focused coping strategies, can facilitate the management of emotions and adversity, which ultimately affects PTG^19^,^20^.

While PTG has been extensively studied in individuals who have survived disasters, such as earthquakes^21^,^22^ and tsunamis^23^,^24^, relatively few studies have investigated PTG in healthcare workers. Healthcare workers are in constant contact with sad, anger, sick and dying people, making them more vulnerable to stress and trauma than individuals in other vocations. Additional stressors include poor sleep quality, an imbalance in effort-reward, working night shifts, workplace accidents, workplace violence, stressful doctor-patient relationships and prolonged work schedules^25^,^26^. Inoue *et al*.^27^ found that 62.7% of psychiatric nurses encountered workplace violence and some of them were diagnosed with PTSD^27^. Approximately 20% of Japanese physicians suffered from insomnia^28^. Moreover, physicians, especially female physicians, are at a higher risk of suicide than the general population^29^. In China, there is a paucity of medical workers. The ratio of doctors to the general population (1:735) in China is much lower than that in Western countries (1:280–1:640)^30^. Therefore, Chinese medical workers may suffer from more serious psychological problems. In fact, 76.9% of Chinese physicians report burnout symptoms^31^. Workplace violence, and symptoms of anxiety and depression are increasingly prevalent among Chinese healthcare workers^32^,^33^,^34^,^35^. Highly challenging work involving human life leads to greater vulnerability to burnout and trauma. While some hospital workers report no direct experience with trauma, the job intensity, extensive contact with patients, empathy, and emotional involvement in patients, can lead to indirect trauma through vicarious traumatization^36^,^37^,^38^. Previous studies revealed that physicians may suffer from posttraumatic stress symptoms after treating a large number of casualties, especially in cases of patient death^39^. Vicarious trauma not only causes harm, but it may also result in PTG in healthcare workers. For example, after exposure to sexual abuse survivors, clinicians report greater spiritual wellbeing^40^. Due to professional characteristics, health care is acknowledged as an essential profession by the public, which is helpful to improve healthcare workers’ self-worth and growth. So nurses demonstrated higher PTG than social workers when working with war victims^41^. Psychotherapists working with trauma survivors have shown positive changes similar to those who experience traumatic events directly^42^.

PTG can be perceived as a protective factor reducing stress and grief. A prior study found that life satisfaction is affected more by PTG than by workplace violence^43^. The development of intervention programs aimed at increasing PTG will benefit quality of life, diminish job burnout and increase care quality in hospital staff. We designed a novel intervention focusing on growth in place of the traditional risk management model. The novel intervention was derived from previous interventions such as Stress Management and Resiliency Training (SMART)^44^; stress management to improve coping skills^45^; an educational course including mindfulness, communication, and self-awareness^46^; an education program based on mindfulness-based stress reduction^47^; Wellness program^48^; educational seminars on compassion fatigue^49^. In addition, Chinese traditional culture ideals were taken into consideration. The purpose of the current study was to explore the impact of the novel intervention on PTG in medical staff.

## Methods

### Participants

According to the convenience sampling method, hospital administrative personnel asked staff with free time to attend a voluntary positive psychological intervention. Medical workers (N = 579) from hospitals in Guilin (n = 199), Shenzhen (n = 249) and Xiangtan (n = 131) participated in the intervention. The sample consisted of 212 males and 367 females with a mean age of 36.31 years (SD = 9.86 years). Among participants, 14 reported that they were suffering from a severe disease; 73 reported a family member or close friend suffered from a severe disease or death; 89 reported financial difficulty, 72 reported difficulty in interpersonal relationships; 2 participants were subjected to domestic violence; 83 suffered from abuse or injury in medical disputes and 93 people reported that they experienced other stressful events.

### Instruments

The Posttraumatic Growth Inventory (PTGI) was used to measure perceived benefits from traumatic events^12^. The Chinese version of the PTGI has been translated into Chinese and modified and has been proven to be a valid tool within the Chinese population^50^,^51^. The PTGI contains 21 items within five categories: relating to others, new possibilities, personal strength, spiritual change and appreciation of life. The Cronbach’s α for the inventory was measured at 0.93 and 0.95 in the current study. Response options range from 0 (no change) to 5 (high degree of change). Higher scores reflect greater perceived benefit.

The Maslach Burnout Inventory (MBI) is the most commonly used standardized measurement tool in the assessment of job burnout^52^. The MBI has been widely used to evaluate Chinese medical staff^53^,^54^. The inventory contains 22 items within three subscales: emotional exhaustion, depersonalization and personal accomplishment. Each subscale is rated on a 7-point scale from 0 (Never) to 6 (Very frequent). Higher scores in emotional exhaustion and depersonalization indicate a higher level of job burnout while a higher score in personal accomplishment indicates a lower level of job burnout. The Cronbach’s α for the MBI in the current study was 0.814.

The Minnesota Satisfaction Questionnaire (MSQ) is considered an effective tool in the measurement of job satisfaction in China^55^. The short version of the MSQ has 20 items within two dimensions: intrinsic satisfaction and extrinsic satisfaction. Individuals respond on a 5-point Likert scale from 1 (very dissatisfied) to 5 (very satisfied). Higher scores represent greater job satisfaction. The Cronbach’s α for the MSQ in the current study was 0.917.

The Chinese short version of the Depression Anxiety and Stress Scales (DASS-C21)^56^ is a revised version of the short Version of the Depression Anxiety Stress Scale^57^ and measures emotional distress. The instrument includes 21 items within three subscales: depression, anxiety and stress. Subjects rate themselves on a 4-point Likert scale ranging from 0 (“did not apply to me at all”) to 3 (“applied to me very much, or most of the time”) to indicate how often they experienced each item during the past week. High scores on the DASS-C21 indicate more severe levels of depression, anxiety and stress. The Cronbach’s α of the DASS-C21 in the current study was 0.939.

### Procedure

The intervention was led by a clinical psychologist and included four phases:

#### Phase 1: Baseline psychological assessment

All participants completed the PTGI, MBI, MSQ and DASS.

#### Phase 2: Psychological health education

This phase consisted of four courses, each lasting approximately thirty minutes. The leader and three other psychologists served as moderators in each course and a psychology postgraduate served as an assistant. A description of the four courses follows: (1) Psychosomatic improvement: The essence of Chinese traditional philosophy was discussed, such as the Five Elements and the Eight Diagrams, to educate participants about life and nature. (2) Psychosomatic self-rating: Depression, anxiety and stress were discussed, including the manifestation of these negative emotions. In addition, the moderator described some common physical symptoms (such as loss of appetite and headaches), cognitive symptoms (such as difficulty concentrating and memory problems), and behavioral symptoms (such as social avoidance and a loss of interest in activities) under occupational stress. The purpose of this course was to help healthcare workers recognize their own psychological issues. (3) Emotion management: Participants learned about the harmful effects of negative emotions and the importance of cognitive appraisal. The participants were taught positive coping strategies to vent negative emotions, such as exercising, paying attention to the positive aspects of the event, and talking with friends. The participants were taught to cultivate an optimistic thinking mode, to increase a positive cognitive appraisal of events, even in adversity. Lastly, participants learned meditation and muscle relaxation techniques to manage stress. (4) Sharing growth. The moderator integrated the growth of the psychological rehabilitation group and shared with all participants. A positive atmosphere along with lively and friendly communication was stressed. All participants were encouraged to share their own experience.

#### Phase 3: Questions and discussion

Participants were invited to ask questions about mental health and work difficulty and a discussion followed.

#### Phase 4: Psychological assessment

The participants completed the PTGI again to assess the effect of intervention on PTG.

The study procedures complied with the current ethical standards for investigation involving human participants in China. All methods were approved by the Second Xiangya Hospital of Central South University Ethics Committee. Informed consent was obtained from all subjects. Our research group registered with the WHO International Clinical Trial Registry Platform on June 17, 2016. Our registration number is ChiCTR-OOC-16008664.

## Results

### Descriptive analyses

Table 1 presents the means, standard deviations and percentages representing the demographic composition of the study sample.

### Correlation analysis

Correlations between pre-intervention scores on the PTG and job burnout, job satisfaction and emotional distress (depression, anxiety and stress) are shown in Table 2. The PTGI total score, and all of the included subscale scores were negatively correlated with emotional exhaustion, depersonalization and emotional distress, and positively correlated with personal accomplishment, intrinsic satisfaction and extrinsic satisfaction.

### Pre-intervention and post-intervention analyses

A paired-samples t-test comparing pre-intervention PTGI scores to post-intervention PTGI scores was carried out to explore the effects of the novel intervention (Table 3). PTGI total score and all subscale scores were significantly higher after intervention (p < 0.001). The aspect of “new possibilities” improved the most with intervention. These results indicate that the positive psychological intervention was effective in improving overall PTG.

### Socio-demographic variables analyses

An independent samples t-test and a variance analysis were performed to explore the impact of socio-demographic variables on intervention efficacy. After intervention, women showed greater improvement than men in the aspect of “new possibilities” (Table 4), nurses showed greater improvement than physicians and than participants in other occupations in “appreciation for life” (Table 5) and those with a bachelor’s degree showed greater improvement in “new possibilities” (Table 6). Place of residence and marriage status did not affect intervention results.

## Discussion

As expected, there was an inverse relationship between PTG and emotional exhaustion, depersonalization, depression, anxiety and stress while there was a positive relationship between PTG and personal accomplishment, intrinsic satisfaction and extrinsic satisfaction. These findings indicate that scores on the PTG are a valid indicator of healthcare workers’ quality of life and work. We found that the positive psychological intervention implemented in the current study significantly improved participants’ PTG. In particular, the subscale of “new possibilities” showed the greatest improvement. Through the intervention, participants had a closer relationship with others and a greater sense of personal strength, increased appreciation for their life and spiritual development, and recognized that there are many new possibilities or paths for their life. Women and nurses showed greater improvement than men and other professionals, respectively. Perhaps due to the more agreeableness in Chinese females^58^ and the professional requirements of nurse, they are better at listening. Therefore them may have learned more skills and knowledge from the intervention, leading to a greater benefit.

After reviewing previous studies that explored the use of interventions on hospital staff^44^,^45^,^46^,^47^,^48^,^49^, along with factors contributing to PTG^13^,^14^,^15^,^16^,^17^,^18^,^19^,^20^, the current study added Chinese traditional cultural ideals as a novel aspect to the intervention provided. Lots of Chinese traditional philosophy deeply discussed human and nature. In modern society, life is busier and work is more intense than in previous eras. The addition of Chinese traditional culture education may affect the thinking patterns of participants who thereby form a more meaningful and valuable narrative about adversity.

Through psychological assessment and psychosomatic self-rating, participants learned about their own psychological issues. An awareness of their vulnerabilities may encourage participant to reach out for social supports which also has been shown to improve PTG^15^,^16^.

Traumatic events inevitably lead to emotional responses. The use of negative strategies, such as smoking and drinking may temporarily increase mood but these behaviors are not good long-term solutions for coping with negative emotions. However, positive coping strategies, such as exercising and healthy eating habits are productive methods in the management or prevention of stress^48^,^49^ and therefore promote the development of PTG. The emotional management phase of our intervention encouraged new skills which helped participants adopt more effective coping strategies and a healthier approach to dealing with traumatic events. Meditation and muscle relaxation techniques are beneficial for increasing emotional stability^46^.

By sharing growth, participants recognized the benefit of the intervention and their positive changes. In addition, participants were encouraged to ask questions and share their thoughts and feelings with other individuals in the same position (hospital staff), who can more readily understand and empathize. This relationship with others can promote a feeling of belongingness and lessen feelings of loneliness, benefiting PTG.

While the results of the current study are beneficial, there are some limitations. First, our sample size was rather small (579) and limited to Guilin, Shenzhen and Xiangtan. Future research should include a greater sample size from a variety of medical personnel in diverse areas of China. Second, the current study did not include a control group. Further studies should compare a group of participants receiving our novel intervention to a group who do not participate in the intervention but rather attend a general lecture. Third, the current study did not separate participants into types of trauma experienced. Future studies should include the Impact of Event scale-Revised and The Secondary Trauma Questionnaire to assess trauma type and severity.

To our knowledge, the current study is the first to use a positive psychological intervention based on the Chinese culture to improve medical workers’ PTG. In conclusion, our novel intervention proved effective, especially in improving the aspect of “new possibilities”. Although preliminary, our results are promising and suggest that psychological intervention can be a useful tool within the medical community to instill positive change. These findings encourage us to pursue this intervention among healthcare workers to improve their quality of life and work.

## Additional Information

**How to cite this article**: Xu, X. *et al*. Effect of Positive Psychological Intervention on Posttraumatic Growth among Primary Healthcare Workers in China: A Preliminary Prospective Study. *Sci. Rep.* **6**, 39189; doi: 10.1038/srep39189 (2016).

**Publisher's note:** Springer Nature remains neutral with regard to jurisdictional claims in published maps and institutional affiliations.

## Figures and Tables

**Table 1:** Demographic information of the study participants.

Variable		N	%	M(SD)	Range
Age				36.31 (9.86)	18–73
Gender	Male	212	36.6		
	Female	367	63.4		
Residence	Rural	405	69.9		
	City	174	30.1		
Occupation	Physician	320	55.3		
	Nurse	153	26.4		
	Other	106	18.3		
Marital Status	Single	163	28.2		
	Married	402	69.4		
	Divorced/widowed	14	2.4		
Highest Educational Level	Vocational School or less	126	21.8		
	Bachelor degree	331	57.1		
	Postgraduate	122	21.1		

**Table 2:** Correlations between scores on the PTGI and other psychology variables.

	Relating to Others	New Possibilities	Personal Strength	Spiritual Change	Appreciation of Life	PTGI Total
EE	−0.167**	−0.269**	−0.225**	−0.246**	−0.243**	−0.257**
DP	−0.181**	−0.166**	−0.174**	−0.163**	−0.195**	−0.201**
PA	0.232**	0.183**	0.295**	0.215**	0.256**	0.266**
Depression	−0.285**	−0.347**	−0.388**	−0.330**	−0.366**	−0.385**
Anxiety	−0.175**	−0.229**	−0.268**	−0.195**	−0.250**	−0.251**
Stress	−0.182**	−0.308**	−0.313**	−0.270**	−0.305**	−0.305**
IS	0.292**	0.313**	0.353**	0.274**	0.305**	0.352**
ES	0.255**	0.337**	0.279**	0.289**	0.286**	0.330**

EE: Emotional exhaustion, DP: Depersonalization, PA: Personal accomplishment, IS: Intrinsic satisfaction, ES: Extrinsic satisfaction **p < 0.01.

**Table 3:** Pre-intervention vs post-intervention scores (M ± SD).

	Pre- Intervention	Post- Intervention	t	Cohen’s d	% Improvement
Relating to Others	22.80 ± 4.23	24.39 ± 4.13	−10.60*	0.38	7.0
New Possibilities	15.79 ± 3.64	17.32 ± 3.18	−12.20*	0.45	9.7
Personal Strength	13.91 ± 2.61	14.61 ± 2.44	−7.66*	0.28	5.0
Spiritual Change	6.33 ± 1.80	6.99 ± 1.58	−10.26*	0.39	10.5
Appreciation of Life	10.10 ± 2.22	10.92 ± 1.99	−11.08*	0.39	8.2
PTGI total scores	68.93 ± 12.69	74.24 ± 11.96	−12.76*	0.43	7.7

^*^p < 0.001.

**Table 4:** Impact of gender on changes in PTGI scores after intervention (M ± SD).

	Male	Female	t	p
Relating to Others	1.42 ± 3.74	1.69 ± 3.54	−0.862	0.389
New Possibilities	1.19 ± 2.83	1.73 ± 3.11	−2.131	0.034
Personal Strength	0.50 ± 2.08	0.81 ± 2.23	−1.649	0.100
Spiritual Change	0.64 ± 1.59	0.68 ± 1.54	−0.248	0.804
Appreciation of Life	0.67 ± 1.65	0.91 ± 1.86	−1.581	0.115
PTGI total scores	4.42 ± 9.73	5.82 ± 10.14	−1.619	0.106

Score change = Score immediately after intervention − Baseline score.

**Table 5:** Impact of occupation on changes in PTGI scores after intervention (M ± SD).

	Physician	Nurse	Other	F	p
Relating to Others	1.66 ± 3.86	1.68 ± 3.31	1.25 ± 3.26	0.593	0.553
New Possibilities	1.50 ± 2.97	1.58 ± 2.86	1.53 ± 3.39	0.033	0.968
Personal Strength	0.76 ± 2.18	0.62 ± 2.21	0.62 ± 2.15	0.299	0.742
Spiritual Change	0.69 ± 1.60	0.65 ± 1.45	0.60 ± 1.60	0.143	0.867
Appreciation of Life	0.87 ± 1.72	1.02 ± 1.78	0.40 ± 1.95	4.045	0.018
PTGI total scores	5.49 ± 10.29	5.55 ± 9.22	4.40 ± 10.27	0.535	0.586

Score change = Score immediately after intervention − Baseline score.

**Table 6:** Impact of education level on changes in PTGI scores after intervention (M ± SD).

	Vocational School or less	Bachelor’s degree	Postgraduate degree	F	p
Relating to Others	1.28 ± 3.51	1.79 ± 3.47	1.38 ± 4.07	1.184	0.307
New Possibilities	1.24 ± 2.90	1.84 ± 2.92	0.99 ± 3.31	4.277	0.014
Personal Strength	0.56 ± 2.18	0.81 ± 2.07	0.52 ± 2.48	1.073	0.343
Spiritual Change	0.67 ± 1.55	0.73 ± 1.60	0.48 ± 1.44	1.190	0.305
Appreciation of Life	0.93 ± 1.92	0.85 ± 1.72	0.64 ± 1.84	0.898	0.408
PTGI total scores	4.69 ± 9.78	6.02 ± 9.59	4.01 ± 11.18	2.120	0.121

Score change = Score immediately after intervention − Baseline score.
